# Quantitative and Fiber-Selective Evaluation for Central Poststroke Pain

**DOI:** 10.1155/2022/1507291

**Published:** 2022-06-06

**Authors:** Jian-Min Chen, Qing-Fa Chen, Zhi-Yong Wang, Guo-Xin Ni

**Affiliations:** ^1^Department of Rehabilitation Medicine, The First Affiliated Hospital of Fujian Medical University, Fujian, China; ^2^Department of Rehabilitation, Fujian Medical University Union Hospital, Fujian, China

## Abstract

The electrophysiological recording can be used to quantify the clinical features of central poststroke pain (CPSP) caused by different lesion locations. We aimed to explore the relationship between clinical features and lesion location in patients with CPSP using the current perception threshold (CPT) approach. Here, patients underwent the standardized CPT measure at five detection sites on both the contralesional and ipsilesional sides, using a constant alternating-current sinusoid waveform stimulus at three frequencies: 2000 Hz, 250 Hz, and 5 Hz. 57 CPSP patients were recruited in this cross-sectional study, including 13 patients with thalamic lesions and 44 patients with internal capsule lesions. Patients with a thalamic lesion had more frequent abnormal A*δ* and C fibers than those with an internal capsule lesion (69.2% versus 36.4%, *p* value = 0.038; 53.8% versus 63.6%, *p* value = 0.038). The patients with internal capsule lesions had more frequent abnormal A*β* fibers than those with thalamic lesions (53.8% versus 63.6%, *p* value < 0.001). The sensory dysfunction in the patients with thalamic lesions was more likely to occur in the upper limbs (i.e., the shoulder (*p* value = 0.027) and the finger (*p* value = 0.040)). The lower limbs (i.e., the knee (*p* value = 0.040) and the toe (*p* value = 0.005)) were more likely to experience sensory dysfunction in the patients with internal capsule lesions. Hyperesthesia was more likely to occur in the thalamic patients, and hypoesthesia was more likely to occur in the patients with internal capsule lesions (*p* value < 0.001). In patients with thalamic lesions, Visual Analogue Scale (VAS) had a positive correlation with 5 Hz CPT on the shoulder (*r* = 0.010, *p* value = 0.005), 250 Hz CPT on the finger (*r* = 0.690, *p* value = 0.009) from the contralesional side, and 2000 Hz CPT on the knee (*r* = 0.690, *p* value = 0.009). In patients with internal capsule lesions, VAS had a positive correlation with 2000 Hz CPT on the knee (*r* = 0.312, *p* value = 0.039) and foot (*r* = 0.538, *p* value < 0.001). In conclusion, the abnormal fiber types, sensory dysfunction territory, and clinical signs of CPSP in thalamic stroke differ from those in internal capsule stroke. Implementation of the portable and convenient CPT protocol may help clarify the locations of different stroke lesions in various clinical settings.

## 1. Introduction

Central poststroke pain (CPSP) is a common complication of stroke [1]. It usually occurs when a stroke happens anywhere in the spinothalamic pathway and its cortical projection [2, 3], but it has also reportedly occurred after capsular hemorrhage, lateral medullary wedge infarction, and cortical infarctions [4].

The cardinal feature of CPSP is pain and sensory dysfunction with onset at or after the stroke [5, 6]. The associated feature and signs of sensory dysfunction depend largely on the lesion location along the neuroaxis [4, 7]. In addition, the anatomical distribution of the sensory dysfunction usually located on the somatotopic arrangement side corresponds to the injured cerebral territory [3, 5]. The detailed mechanism underlying CPSP is still under investigation, but its diverse clinical features may be specifically linked to the functional impairment of sensory fibers [8].

Most strokes cause CPSP damage to not only the central nervous system but also the entire peripheral nerve or the sensory pathways [4]. Sensory processing is the precise integration that requires a balanced interaction between the primary afferent fibers, the spinal cord, the brainstem, and the thalamocortical circuits [3]. Therefore, quantifying the neuroexcitatory effects of sensory fibers using the current perception threshold (CPT) may make possible the exploration of the nonnociceptive pathways (the large A*δ* fibers in the periphery) and the nociceptive transmission system (fibers A*δ* and C in the periphery of the spinothalamic tract system at the central level) [5, 6, 9, 10]. The ability to use reproducible stimuli to estimate sensory thresholds and differentiate symptoms (hyperesthesia and hypoesthesia) may also enhance the technology to make it a powerful method of investigating somatosensory disturbances of various modalities [2, 11].

The Neurometer CPT (Neurotron, Baltimore, MD, USA) is an electrophysiological recording apparatus that selectively measures the thresholds of three classes of afferent fibers by applying three different sinusoidal frequencies (2000 Hz, 250 Hz, and 5 Hz) at various intensities [12, 13]. This study was aimed at evaluating the sensory transmission in CPSP patients using the CPT approach and exploring the relationship between the changes in the perceptual threshold in fibers and lesion location.

## 2. Method

We included 57 patients with CPSP due to a recent first attack of unilateral stroke who were consecutively admitted to the Department of Rehabilitation Medicine of the First Affiliated Hospital of Fujian Medical University from January 2017 to December 2018 in this cohort study. The study was approved by the ethics committee of the First Affiliated Hospital of Fujian Medical University (approval number [2018]102). The entire study design and procedures were performed in accordance with the Declaration of Helsinki. The patients' informed consent was obtained before they participated in this study. The chictr.org identifier is ChiCTR1800019318.

**Figure 1:**
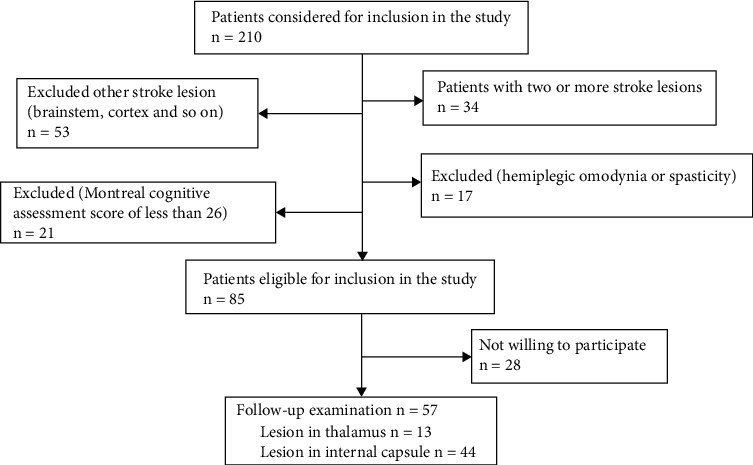
Flowchart of the study.

The inclusion criteria were a solitary and chronic stroke lesion in the internal capsule or thalamus confirmed through magnetic resonance diffusion and computerized tomography and contralateral somatosensory symptoms developed within 6 months of stroke [2, 14]. Our exclusion criteria were stroke lesions in other parts of the brain; two or more stroke lesions; other nonstroke lesions in the brain; any other musculoskeletal, internal, or neurological disorder that can potentially confound the etiology of their pain syndrome (i.e., including other entities of poststroke pain such as hemiplegic omodynia or spasticity); and a Montreal Cognitive Assessment score of less than 26 (Figure 1). The complete neurologic history and clinical examination including sensory testing was performed within three days after the patient's hospital admission by a trained clinician and a rehabilitation physician who was blinded to the experiment design (ZYW and QFC). The neurologic history included pain duration, localization, drug management, rehabilitation therapy, and so on. The sensory testing carried out included touch, pinprick, pressure, vibration, cold, and heat sensation [6, 21].

### 2.1. Current Perception Threshold

**Figure 2:**
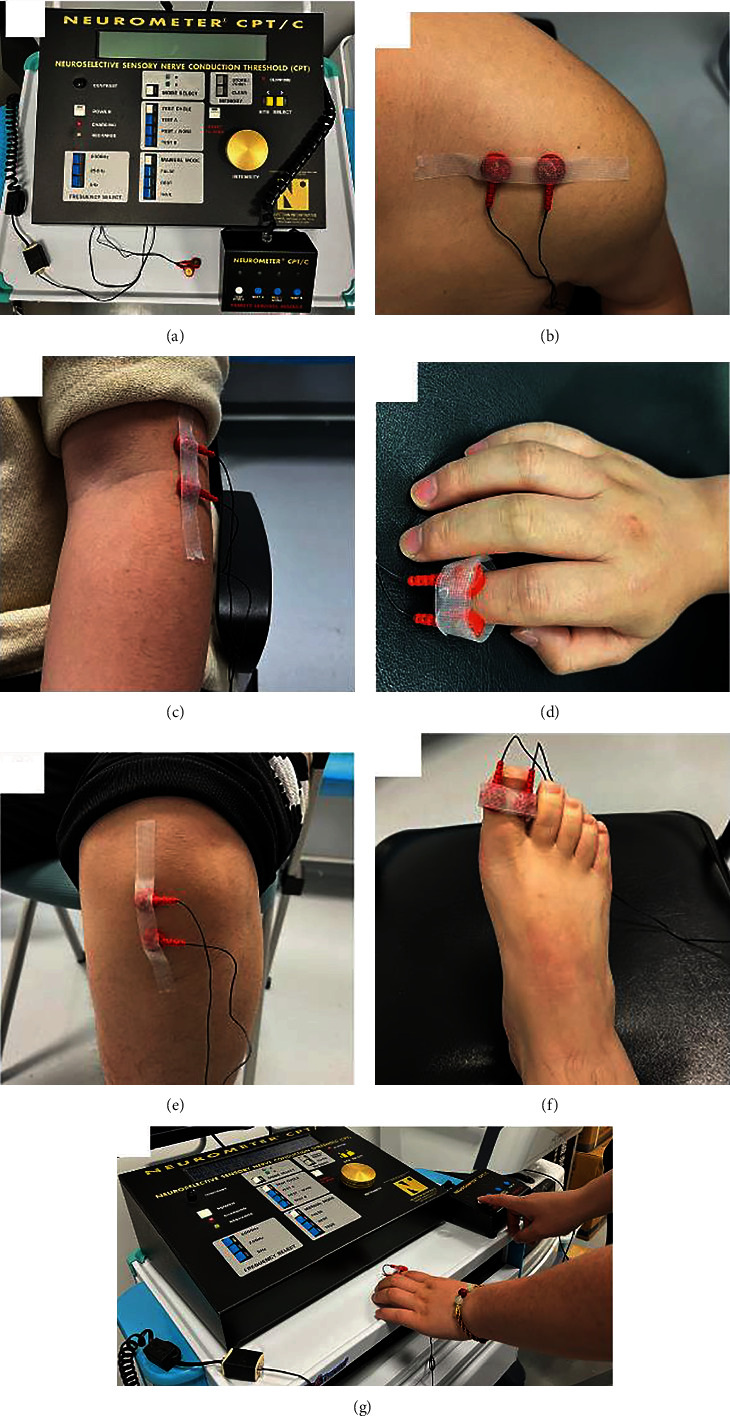
Methods of CPT measurement: (a) image of the Neurometer® CPT device; (b–f) testing site and the placement of the electrode; (g) a patient was tested using the automated forced choice CPT determination mode.

The current perception threshold was conducted in a quiet room using an automated value procedure measured as described in previous studies [13]. Participants' skin was prepared with a skin preparation paste in which a pair of separate round gold-plated (1 cm in diameter) electrodes were coated with hypoallergenic electrode gel and then taped to the test site. Following this, a painless transcutaneous electrical stimulus was administered to their skin at symptomatic sites—on the upper or lower limbs contralateral to the stroke hemisphere, including the shoulder, the upper limb proximal (the elbow), the index finger, the lower limbs proximal (the knee), and the foot (the big toe). The intrasubject control site was the same area on the ipsilesional side. The placement of the electrode is shown in Figure 2.

The CPT test began with the “intensity alignment mode.” The alternating constant current stimulus intensity was increased from 0.01 mA to a maximum of 9.99 mA until the patients reported a sensation at the site of the electrodes. Then, the stimulus was applied at decreasing intensities until it was no longer detected, within a range of ±0.05 mA, after which it was turned off. Next, to measure the CPT value of the patients, which is the minimum degree of painless transcutaneous electrical stimulus, the “automated forced choice CPT determination mode” was performed. Each subject was presented with a series of forced-choice tests, which consisted of randomly generated pairs of real and false (placebo) stimuli presented as test A and test B, which were separated by a rest period. Based on their response, the sensory nerve conduction threshold (sNCT) device automatically adjusted the output level of the stimulus and randomly generated a new testing order for the next pair of tests in the series. Randomly placed double-false tests were also presented to assist in monitoring the subject responses for consistency and accuracy. The value was defined as the average of the minimum intensity of the stimulus consistently detected. This testing sequence was repeated for three frequencies (2000 Hz, 250 Hz, and 5 Hz) before moving to the next site. For each frequency, the lowest electric stimulus intensity that was perceived consistently at each detection point was converted automatically to a CPT value (1 CPT unit = 0.01 mA) using Neurometer® CPT (Neurotron, Baltimore, MD, USA). The total test time for each site was no more than 15 min.

The normative CPT values were obtained from previous clinical trials [15–18] and were earlier incorporated into the system's data analysis software (Neurotron, Baltimore, MD, USA, http://www.neurotron.com). CPT abnormalities are distributed in a two-sided distribution. We defined hyperesthesia when a CPT value is below the normal range (as less current was needed to evoke a response), and dysesthesia was defined as CPTs above the normal range (as the greater current was needed to evoke a response) [18, 19].

### 2.2. Statistical Analysis

Qualitative data were uttered as numbers and percentages. The distribution of quantitative data was evaluated with a normality test (one-sample Kolmogorov-Smirnov test). Normally distributed variables were presented as the mean (standard deviation) and nonnormally distributed variables as the median (interquartile range). We used Wilcoxon's signed-rank test to examine the statistical differences in the CPT measures between the contralesional and ipsilesional sides. Fisher's exact test was performed to determine the correlation of the lesion location to the severity, the distribution of the CPSP, and the class of the abnormal sensory fiber on the contralesional side. The Spearman rank correlation was used to test for a significant association between the Visual Analogue Scale (VAS) score and the CPT value of test sites. McNemar's test was conducted to determine the correlation between clinical characteristics and CPT findings. A *p* value of 0.05 or less was considered statistically significant. All statistics were performed using the SPSS software (version 23.0, IBM Corporation, Armonk, NY, USA).

## 3. Results

### 3.1. Demographic and Clinical Characteristics

We included 57 CPSP patients in this study (median age: 54 years (range, 47-64); women, 14 (24.6%)). CPSP developed after a median duration of 1 month (range, 0.6-2 months).  Table 1 presents the demographic and clinical characteristics of the participants. Of the patients, 42.11% (*n* = 24) and 57.89% (*n* = 33) suffered from cerebral hemorrhage and infarction, respectively, with a right/left stroke hemisphere ratio of 37/20. The lesions were located at the thalamus in 13 (22.81%) patients and the internal capsule in 44 (77.19%) patients. The severity of sensory dysfunction was indicated by the VAS score, and the median score was 6 (5-8) in patients with internal capsule lesions and 6 (4-7) in the patients with thalamic lesions.

### 3.2. Sensory Testing

According to clinical examination findings, 25 patients with internal capsule lesions had abnormal sensations in the lower limb, and 14 had abnormal sensations in the upper limb. In addition, abnormal sensation of the upper limb and lower limb was present in 8 and 3 patients with the thalamic lesion, respectively. Two patients with thalamic lesions and 5 patients with internal capsule lesions were hemibody (Details in supplementary).  Table 2 shows that 11 patients had sensory gain (7 with thalamic lesions and 4 with internal capsule lesions) and 46 had sensory loss (6 with thalamic lesions and 40 with internal capsule lesions).

### 3.3. CPT Parameters

#### 3.3.1. Sensory Sensitivity in CPSP Patients

**Figure 3:**
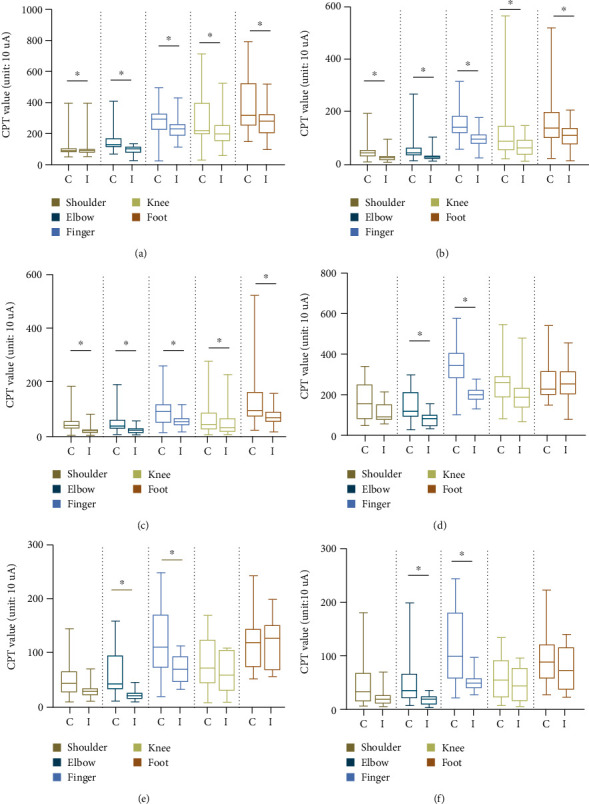
Comparison between CPT values from the contralesional side and ipsilesional side. (a–c) CPT values of patients with internal capsule lesions (*n* = 44). (d–f) CPT values of patients with thalamic lesions (*n* = 13). *p* value refers to the results of Wilcoxon's signed-rank test (^∗^*p* < 0.05). C: site on the contralesional side; I: site on the ipsilesional side.

The CPT test was performed with three frequencies at 10 detection sites on both sides.  Figure 3 shows that CPSP patients exhibited a considerably lower sensory sensitivity at each frequency on the site of the contralesional side than that on the ipsilesional side. Specifically, statistical differences were revealed in all detection sites for the two sides in patients with internal capsule lesions. However, the difference was seen only in the elbow and hand in those with thalamus lesions.

### 3.4. Comparison between Lesion Location and Type of Abnormal Fiber

**Figure 4:**
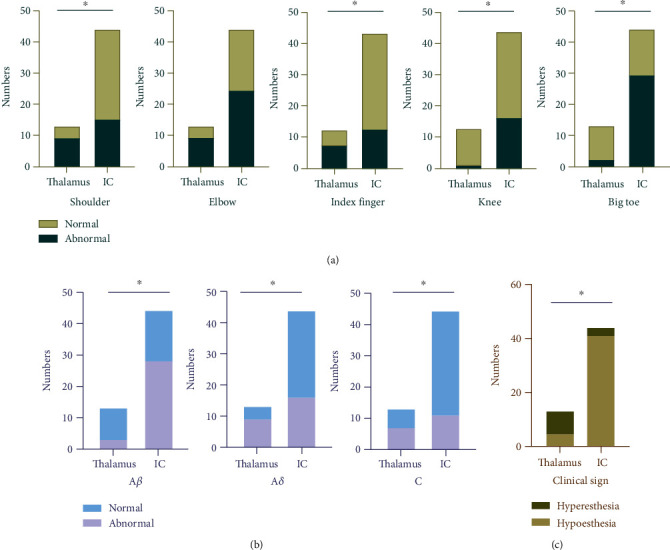
The distribution of the sensory disturbance, the class of the abnormal sensory fibers, and the clinical sign in CPSP patients: (a) numbers of patients with normal and abnormal CPT values in each test site; (b) numbers of patients with normal and abnormal CPT values in each sensory fiber; (c) numbers of patients with hypoesthesia and hyperesthesia. *p* value refers to the results of Fisher's exact test (^∗^*p* < 0.05).

Table 3 and Figure 4 show that the type of abnormal fibers was correlated with the lesion location. Patients with thalamic lesions had more frequent abnormal A*δ* (9 (69.23%) vs. 16 (36.36%), *p* value = 0.038) and C (7 (53.85%) vs. 11 (25.00%), *p* value = 0.038) fibers than those with internal capsule lesions. The A*β* fibers were abnormal in 3 (23.08%) patients with thalamic lesions and in 28 (63.64%) patients with internal capsule lesions (*p* value = 0.011).

### 3.5. Comparison between Lesion Location and Sensory Dysfunction Territory

The sensory dysfunction territory was correlated with the location of the stroke. The distribution of the sensory dysfunction was hemibody. The territories were mostly in multiple areas, including, among the 13 patients with thalamic lesions, the shoulder in 9 patients, the elbow in 9, the finger in 8, the toe in 2, and the knee in 1 and, among the 44 patients with internal capsule lesions, the toe in 29 patients, the elbow in 24, the knee in 16, the shoulder in 15, and the finger in 13. The sensory dysfunction in the patients with thalamic lesions was more likely to occur in the shoulder (*p* value = 0.027) and in the finger (*p* value = 0.040), whereas in the patients with internal capsule lesions, it was more likely to occur in the lower limbs, i.e., in the knee (*p* value = 0.040) and in the toe (*p* value = 0.005). The patients with thalamic lesions had a more frequent abnormality of CPT value in the elbow (9 (69.23%) vs. 24 (54.55%), *p* value = 0.267) than the patients with internal capsule lesions, although this difference was not statistically significant. The details are summarized in Table 3 and Figure 4.

### 3.6. Comparison between Lesion Location and Clinical Signs

The clinical signs of CPSP were significantly related to the location of the stroke. Of the 13 patients with thalamic lesions, 4 (30.77%) patients had CPT values below the normal range, which suggests hyperesthesia, and 9 (69.23%) patients had CPT values above the normal range, which suggests hypoesthesia. Of the 44 patients with internal capsule lesions, however, 3 (6.82%) patients had a decrease in their CPT values and 41 (93.18%) had an increase (*p* value < 0.001). The details are summarized in Table 3 and Figure 4.

### 3.7. The Relationship between Clinical Characteristics and CPT Findings

The CPT value at 5 Hz of the shoulder from the contralesional side in patients with thalamic lesions exhibited a significant positive correlation with the VAS score (*r* = 0.010, *p* value =0.005), as did the 250 Hz of the finger (*r* = 0.690, *p* value =0.009). The CPT value at 2000 Hz of the knee and foot from the contralesional side in patients with internal capsule lesions exhibited a significant positive correlation with VAS score (knee: *r* = 0.312, *p* value = 0.039; foot: *r* = 0.538, *p* value < 0.001). Furthermore, the number of clinical signs (sensory gain and sensory loss) by clinical examination was related to the CPT findings (hyperesthesia and hypoesthesia) in patients with thalamic lesions (*p* value = 0.687) and patients with internal capsule lesions (*p* value = 1.0), respectively.

## 4. Discussion

In this study, we investigated the sensory disturbance objectively by using the CPTs of patients with CPSP. We found that CPSP has a wide clinical spectrum that ranges from a limited to a widespread topography of hyperesthesia to hypoesthesia because of thalamic and internal capsule lesions with abnormal sensory fibers. The types of abnormal fibers, sensory dysfunction territories, and the clinical signs of CPSP differed between thalamic and internal capsule strokes.

The nature of the associated neurological signs and features of CPSP depends largely on the lesion location along the neuroaxis [20]. In this study, abnormal A*β* fibers were correlated with the location of the lesion in the CPSP patients. The patients with internal capsule lesions had a higher frequency of abnormal A*β* fibers. The defective threshold of the A*β* fibers was associated with changes in the large-fiber somatosensory pathway [13]. The internal capsule patients usually had an abnormal touch, vibration, and joint position sensations [4, 21], which were associated with changes in their somatosensory evoked potential (SEP) [22]. The cortical SEP is the response of the primary sensory cortex mediated through peripheral large-fiber afferents, the dorsal column medial lemniscus, and the internal capsule [23]. Some evidence suggests that the dorsal column medial lemniscus sensory pathway is involved in the generation of neuropathic sensory dysfunction in internal capsule patients. Another explanation of the higher frequency of abnormal A*β* fibers in internal capsule patients is the association of the dysfunction of the medial lemniscus pathway to motor intracortical inhibition. The internal capsule patients with an incomplete corticospinal tract expressed an increase in motor intracortical inhibition [22]. This study further provided evidence that sensory dysfunction usually develops as CPSP patients become weaker. Weakness motor dysfunction was the presenting symptom of stroke in most of the internal capsule patients (42/44), but it was not common among the thalamic patients (2/13). Therefore, due to the negative impact of the dorsal column medial lemniscus and weakness, patients with internal capsule lesions were more likely to present abnormal A*β* fibers than thalamic patients. In addition, this study showed a significant correlation between abnormal nociceptive (A*δ* and C) fibers and the lesion location. The incidence of A*δ* and C fiber abnormalities among the thalamic patients was higher than that among the IC patients. Gritsch et al. found, through behavioral analysis after stereotactical lesioning of the thalamus, that the sensory changes in their model mainly manifested in mechanical and temperature dysfunction [24]. This phenomenon might have been caused by the transmission of pinprick perception and temperature sensitivity by the spinothalamic tract (STT) [25, 26]. In cases in which the lesion involves the STT, interruption of such transmission due to the stroke will result in necrosis, which will, in turn, result in profound nociceptive transmission system abnormalities [24]. Therefore, thalamic lesions tend to produce marked nociceptive CPT abnormalities.

Various studies have revealed that the distribution of sensory dysfunction territory depends to some extent on the lesion location [21, 27]. The distribution of the sensory dysfunction in discretely localized body parts, such as the arms, legs, trunk, and face, in terms of frequency, has a hemibody pattern with predominant limb extremities [4, 28]. Based on the diffusion tensor imaging findings of Mohamed et al. [29], they suggest that these clinical manifestations are directly related to the damage of the neural connections within the spinothalamocortical pathway. Thus, in our study, the CPSP patients with thalamic lesions exhibited the aforementioned clinical features. These patients exhibited a higher frequency of sensory abnormalities in their upper limbs (shoulders and fingers) after their thalamic lesions. However, Kim [30] found that in the majority of patients with internal capsule lesions, the CPSP was distinctly more severe in the leg than in the arm, and in many instances, the sensory dysfunction was limited to the lower leg or even to the foot area. In our study, our results are consistent with previous observations that patients with internal capsule lesions tend to report a higher frequency of sensory dysfunction in their lower limbs (knees and toes). The mechanism of leg-dominant sensory dysfunction in internal capsule CPSP remains speculative. In primates, the face, arm, and leg areas are arranged in a medial-lateral direction in the ventral posterior nucleus of the thalamus [20, 30]. A similar topography appears to be preserved in the thalamocortical sensory radiation. Considering the neuroradiologic data of the patients with an internal capsule lesion in this study, the lesion locations might have involved the sensory tracts that originate from the most dorsolateral portion of the ventral posterior nucleus, which would explain the leg-dominant sensory symptoms. Whatever the mechanism may be, the rare association of the leg-dominant pattern of sensory dysfunction in CPSP patients with the occurrence of stroke lesions at other locations appears to be one of the characteristics of CPSP in patients with internal capsule lesions [30, 31].

According to previous studies, the diagnosis of CPSP is one of exclusion, as there are no pathognomonic features of this syndrome [2, 23, 32]. The electrophysiological recording, sensory testing, or clinical scales are also used to assist in the diagnosis of CPSP [21, 33, 34]. Several semiquantitative scales have been used to evaluate the severity of CPSP, including the VAS and daily pain rating scale [14, 35]. In this study, the clinical scale was conducted by VAS. As mentioned earlier, the sensory dysfunction in the patients with thalamic lesions was more likely to occur in the A*β* fibers of the shoulder and finger, whereas in the patients with internal capsule lesions, the sensory dysfunction was more likely to occur in the A*δ* and C fiber of the lower limbs. In these frequencies of sites referred to above, the CPT values show a positive correlation with the severity of CPSP valued by the VAS score of patients. It seems that the CPT test appeared to be an objective method to quantify the degree of sensory dysfunction in patients of CPSP.

Recent studies have suggested that CPSP may be characterized by positive and negative symptoms. On the one hand, positive symptoms, such as hyperesthesia, often reflect abnormal excitability of the nervous system [36]. In our study, more of the patients with thalamic lesions manifested hyperesthesia. Lesion-induced hyperexcitability has been suggested as playing an important role in the pathophysiology of thalamic CPSP. An interruption of the STT in rats, primates, and humans has been shown to cause hyperactivity in the thalamic lesion. This hyperactivity has also been described with the use of single-photon emission computed tomography and positron emission tomography in thalamic CPSP patients who were experiencing hyperesthesia [24]. The negative symptoms, also known as hypoesthesia, are usually the result of axon/neuron loss. In literature, hypoesthesia at the detection site suggests that a higher current threshold or stimulus intensity is required to evoke a perception in patients. Conversely, a lower current threshold or stimulus intensity can evoke a response that can lead to sensory gain (hyperesthesia) [11, 19]. In this study, hypoesthesia was more frequent in internal capsule patients than in thalamic patients. Somatosensory disconnection has been considered a possible clue to the mechanism of CPSP related to internal capsule stroke. Internal capsule lesions interrupt sensory pathways between the thalamus and the cortex, and their sequelae may present examples of differentiation [37]. In our study, sensory testing revealed sensory gain in 7 and sensory loss in 6 out of 13 patients with thalamic lesions which correlated with corresponding hyperesthesia and hypoesthesia on CPT findings in 9 and 4 patients, respectively. Also, the sensory testing (sensory gain in 4 and sensory loss in 40) was also correlated with the CPT findings (hyperesthesia in 3 and hypoesthesia in 41) in these 44 patients with internal capsule lesions. Therefore, unlike sensory testing influenced by the subjective nature of sensation [19], CPT studies also provide an opportunity to identify positive and negative symptoms.

Given the above considerations, CPTs could help clarify and quantify the clinical features of CPSP caused by different stroke lesion locations and can provide a simple representation of the underlying pathophysiological mechanisms. The results of this study suggest that in the absence of imaging examination (e.g., in outpatient and rehabilitation clinics and community health centers), CPT values may be able to help infer the location of the lesion in CPSP patients. In addition, electrophysiological studies can provide prognostic information on the likelihood of the development of CPSP in several cases [8]. However, most electrophysiological studies have a high cost, require long-term testing, and are unavailable in many treatment settings [34]. The current CPT responds to the need for a convenient testing protocol that enables a thorough evaluation of CPSP patients in various settings [19]. It is noteworthy that changes in the CPT values may indicate changes in the condition of patients. If the CPT value is reversed, it may also be the result of medication or rehabilitation intervention [10, 20].

## 5. Strength and Limitations

Collectively, this study for the first time evaluates the neuroexcitatory characteristics of sensory fibers in patients with CPSP with detailed quantitative testing. The appealing properties of CPT make it a potentially attractive candidate for larger, clinic-based studies. The findings from this study appear promising, and further investigation of CPTs has the potential to contribute to advancing CPSP research. Nevertheless, multiple limitations should be considered when interpreting the results of this study. First, our findings were limited by the small sample size and its failure to assess full CPSP lesions (e.g., we did not enroll any patient with medullary infarction or cortical infarction). Second, we were unable to control for or systematically evaluate the effects of patients' medication regimens on their sensory responses. Third, the CPT test was performed in a laboratory setting. The characteristics of the equipment used should be further investigated in less controlled settings and with larger samples to assess its reproducibility under an array of clinical conditions. However, the sensory testing in this study was conducted with simple tools at poorly bedside rather than in the laboratory. Finally, this study used normative CPT values as references without recruiting healthy volunteers, which is beyond the scope of this study of merely gathering pilot data to support the need for continuing research within this field in more clinical settings and with larger sample sizes.

## 6. Conclusions

The diverse clinical features of CPSP may be specifically linked to functional impairment of sensory fibers. The assessment of sensory fibers using CPTs can help clarify the clinical manifestations of CPSP caused by different stroke lesion locations. The findings from this study appear promising, and additional investigations of this CPT test have the potential to contribute to advancing CPSP research and provide fundamental information for clinical practice.

## Figures and Tables

**Table 1:** Demographic and clinical characteristics of the study population (*n* = 57).

Variable	CPSP patients
Female/male (*n*)	14/43
Median age (y)	54 (47-64)
Median (mean) time from stroke onset (mo)	0.86 (0.6-2)
Lesion location (m)	
Right side/left side	37/20
Thalamus	13
Internal capsule	44
Type of stroke (*n*)	
Hemorrhage	24
Infarction	33
Score of VAS	
Thalamus	6 (5-8)
Internal capsule	6 (4-7)

*n*: number; y: year; mo: month.

**Table 2:** Correlation between clinical characteristics and CPT findings.

	Sensory testing (*n*)		*p* value
	Sensory gain	Sensory loss	
CPT findings (*n*)			
Internal capsule			
Hypoesthesia	5	4	0.607
Hyperesthesia	2	2	
Thalamus			
Hypoesthesia	2	1	1
Hyperesthesia	2	39	

*n*: number. Data are number of patients. *p* value refers to the results of McNemar's test. Significance level at *p* < 0.05.

**Table 3:** Comparison between the location of the stroke lesion and the distribution of the sensory disturbance, the class of the abnormal sensory fibers, and the clinical sign.

Variable	Thalamus (*n* = 13)	Internal capsule (*n* = 44)	*p* value
Test site			
Shoulder			
Abnormal	9 (69.23%)	15 (34.09%)	0.027
Normal	4 (30.77%)	29 (65.91%)	
Elbow			
Abnormal	9 (69.23%)	24 (54.55%)	0.269
Normal	4 (30.77%)	20 (45.45%)	
Index finger			
Abnormal	8 (61.54%)	13 (29.55%)	0.040
Normal	5 (38.46%)	31 (70.45%)	
Knee			
Abnormal	1 (7.69%)	16 (36.36%)	0.040
Normal	12 (92.31%)	28 (63.64%)	
Big toe			
Abnormal	2 (15.38%)	29 (65.91%)	0.005
Normal	11 (84.62%)	15 (34.09%)	
Fiber type			
A*β*			
Abnormal	3 (23.08%)	28 (63.64%)	0.011
Normal	10 (76.92%)	16 (36.36%)	
A*δ*			
Abnormal	9 (69.23%)	16 (36.36%)	0.038
Normal	4 (30.77%)	28 (63.64%)	
C			
Abnormal	7 (53.85%)	11 (25.00%)	0.038
Normal	6 (46.15%)	33 (75.00%)	
Clinical sign			
Hypoesthesia	4 (30.77%)	41 (93.18%)	<0.001
Hyperesthesia	9 (69.23%)	3 (6.82%)	
VAS score	6 (5-8)	6 (4-7)	0.30

Hyperesthesia is indicated when the upper limit of the normal range is exceeded. Hypoesthesia is indicated when the lower limit of the normal range is exceeded. *n*: number. Data are number of patients (%). *p* value refers to the results of Fisher's exact test. Significance level at *p* < 0.05.

## Data Availability

The data that support the findings of this study are available from the corresponding author (GXN) upon reasonable request.

## Supplementary Materials

Supplementary Materials (1) Median CPT parameters of the contralesional side and ipsilesional side in CPSP patients. (2) Pain symptomatology of central poststroke pain. (3) STROBE Checklists.

## References

[1] Franzini A., Messina G., Levi V. (2020). Deep brain stimulation of the posterior limb of the internal capsule in the treatment of central poststroke neuropathic pain of the lower limb: case series with long-term follow-up and literature review. *Journal of Neurosurgery*.

[2] Osama A., Abo Hagar A., Elkholy S., Negm M., Abd El-Razek R., Orabi M. (2018). Central post-stroke pain: predictors and relationship with magnetic resonance imaging and somatosensory evoked potentials. *The Egyptian Journal of Neurology, Psychiatry and Neurosurgery*.

[3] Wan L., Li Z., Liu T. (2020). Epoxyeicosatrienoic acids: emerging therapeutic agents for central post-stroke pain. *Pharmacological Research*.

[4] Kumar B., Kalita J., Kumar G., Misra U. K. (2009). Central poststroke pain: a review of pathophysiology and treatment. *Anesthesia and Analgesia*.

[5] Klit H., Finnerup N. B., Andersen G., Jensen T. S. (2011). Central poststroke pain: a population-based study. *Pain*.

[6] Mhangara C. T., Naidoo V., Ntsiea M. V. (2020). The prevalence and management of central post-stroke pain at a hospital in Zimbabwe. *Malawi Medical Journal*.

[7] Treister A. K., Hatch M. N., Cramer S. C., Chang E. Y. (2017). Demystifying poststroke pain: from etiology to treatment. *PM & R : The Journal of Injury, Function, and Rehabilitation*.

[8] Garcia-Larrea L., Hagiwara K. (2019). Electrophysiology in diagnosis and management of neuropathic pain. *Revue Neurologique (Paris)*.

[9] Ogawa T., Kimoto S., Nakashima Y. (2017). Measurement reliability of current perception threshold and pain threshold in parallel with blood sampling. *Clinical and Experimental Dental Research*.

[10] Chen Y., Mao C. J., Li S. J. (2015). Quantitative and fiber-selective evaluation of pain and sensory dysfunction in patients with Parkinson's disease. *Parkinsonism & Related Disorders*.

[11] Griffith K. A., Couture D. J., Zhu S. (2014). Evaluation of chemotherapy-induced peripheral neuropathy using current perception threshold and clinical evaluations. *Support Care Cancer*.

[12] Cho Y. W., Kang M. S., Kim K. T. (2017). Quantitative sensory test for primary restless legs syndrome/Willis-Ekbom disease using the current perception threshold test. *Sleep Medicine*.

[13] Kodama M., Aono K., Masakado Y. (2009). Changes in sensory functions after low-frequency repetitive transcranial magnetic stimulation over the motor cortex. *The Tokai Journal of Experimental and Clinical Medicine*.

[14] Kalita J., Kumar B., Misra U. K., Pradhan P. K. (2011). Central post stroke pain: clinical, MRI, and SPECT correlation. *Pain Medicine*.

[15] Takekuma K., Ando F., Niino N., Shimokata H. (2000). Age and gender differences in skin sensory threshold assessed by current perception in community-dwelling Japanese. *Journal of Epidemiology*.

[16] Kim H. S., Kho H. S., Kim Y. K., Lee S. W., Chung S. C. (2000). Reliability and characteristics of current perception thresholds in the territory of the infraorbital and inferior alveolar nerves. *Journal of Orofacial Pain*.

[17] Ro L. S., Chen S. T., Tang L. M., Hsu W. C., Chang H. S., Huang C. C. (1999). Current perception threshold testing in Fabry's disease. *Muscle & Nerve*.

[18] Doi D., Ota Y., Konishi H., Yoneyama K., Araki T. (2003). Evaluation of the neurotoxicity of paclitaxel and carboplatin by current perception threshold in ovarian cancer patients. *Journal of Nippon Medical School*.

[19] Yin H., Liu M., Zhu Y., Cui L. (2018). Reference values and influencing factors analysis for current perception threshold testing based on study of 166 healthy Chinese. *Frontiers in Neuroscience*.

[20] Bowsher D., Leijon G., Thuomas K. A. (1998). Central poststroke pain: correlation of MRI with clinical pain characteristics and sensory abnormalities. *Neurology*.

[21] Misra U. K., Kalita J., Kumar B. (2008). A study of clinical, magnetic resonance imaging, and somatosensory-evoked potential in central post-stroke pain. *The Journal of Pain*.

[22] Tang S. C., Lee L. J., Jeng J. S. (2019). Pathophysiology of central poststroke pain: motor cortex disinhibition and its clinical and sensory correlates. *Stroke*.

[23] Klit H., Finnerup N. B., Jensen T. S. (2009). Central post-stroke pain: clinical characteristics, pathophysiology, and management. *Lancet Neurology*.

[24] Gritsch S., Bali K. K., Kuner R., Vardeh D. (2016). Functional characterization of a mouse model for central post-stroke pain. *Molecular Pain*.

[25] Kim J. H., Greenspan J. D., Coghill R. C., Ohara S., Lenz F. A. (2007). Lesions limited to the human thalamic principal somatosensory nucleus (ventral caudal) are associated with loss of cold sensations and central pain. *The Journal of Neuroscience: The Official Journal of the Society for Neuroscience*.

[26] Henry J. L., Lalloo C., Yashpal K. (2008). Central poststroke pain: an abstruse outcome. *Pain Research & Management*.

[27] Merkies I. S., Faber C. G., Lauria G. (2015). Advances in diagnostics and outcome measures in peripheral neuropathies. *Neuroscience Letters*.

[28] Kim J. S. (1998). Delayed-onset ipsilateral sensory symptoms in patients with central poststroke pain. *European Neurology*.

[29] Seghier M. L., Lazeyras F., Vuilleumier P., Schnider A., Carota A. (2005). Functional magnetic resonance imaging and diffusion tensor imaging in a case of central poststroke pain. *The Journal of Pain*.

[30] Kim J. S. (2003). Central post-stroke pain or paresthesia in lenticulo-capsular hemorrhages. *Neurology*.

[31] Kim J. S. (1999). Lenticulocapsular hemorrhages presenting as pure sensory stroke. *European Neurology*.

[32] Greenspan D. J., Ohara S., Sarlani E., Lenz A. F. (2004). Allodynia in patients with post-stroke central pain (CPSP) studied by statistical quantitative sensory testing within individuals. *Pain*.

[33] Baron R., Binder A., Wasner G. (2010). Neuropathic pain: diagnosis, pathophysiological mechanisms, and treatment. *Lancet Neurology*.

[34] Koulouris A. E., Edwards R. R., Dorado K. (2020). Reliability and validity of the Boston bedside quantitative sensory testing battery for neuropathic pain. *Pain Medicine*.

[35] Jin Y., Xing G., Li G. (2015). High frequency repetitive transcranial magnetic stimulation therapy for chronic neuropathic pain: a meta-analysis. *Pain Physician*.

[36] Hassaballa D., Harvey R. L. (2020). Central pain syndromes. *NeuroRehabilitation*.

[37] Schott G. D. (1996). From thalamic syndrome to central poststroke pain. *Journal of Neurology, Neurosurgery, and Psychiatry*.

