# Supplementary material for: Quantitative and Fiber-Selective Evaluation for Central Poststroke Pain
Source: Neural Plast. 2022 Jun 6;2022:1507291. doi: 10.1155/2022/1507291 (PMC9192306; doi:10.1155/2022/1507291)
Supplement: Supplementary Materials — (1) Median CPT parameters of the contralesional side and ipsilesional side in CPSP patients. (2) Pain symptomatology of central poststroke pain. (3) STROBE Checklists. [file 1507291.f1.zip › Supplement 1.docx]

**Table 1. Comparison between CPT values from contralesional side and ipsilesional side**

| Lesion | Current  frequency | Side | Test site | | | | |
| --- | --- | --- | --- | --- | --- | --- | --- |
|  |  |  | Shoulder | Elbow | Finger | Knee | Foot |
| Thalamus  n=13 | 2000Hz | Con | 158 (81, 256) | 120 (93, 220) | 330 (239, 410) | 267 (186, 301) | 230 (199, 322) |
|  |  | Ips | 93 (74, 157) | 85 (47, 108) | 200 (175, 245) | 191 (136, 242) | 257(200, 322) |
|  |  | P-value | 0.153 | 0.022 | 0.003 | 0.91 | 0.762 |
|  | 250Hz | Con | 45 (27, 67) | 46 (32, 96) | 110 (71, 172) | 72 (42, 126) | 120 (73, 144) |
|  |  | Ips | 30 (23, 35) | 22 (15, 29) | 71 (44, 93) | 60 (30, 107) | 128 (67, 152) |
|  |  | P-value | 0.091 | 0.007 | 0.39 | 0.311 | 0.579 |
|  | 5Hz | Con | 35 (16, 70) | 37 (21, 68) * | 99 (59, 181) | 56 (23, 92) | 90 (57, 123) |
|  |  | Ips | 20 (12, 29) | 20 (10, 26) | 50 (40, 60) | 45 (18, 80) | 73 (37, 118) |
|  |  | P-value | 0.101 | 0.007 | 0.10 | 0.511 | 0.362 |
| Internal capsule  n=44 | 2000Hz | Con | 128 (103, 169) | 120(100, 132) | 287 (218, 328) | 222(193, 376) | 310 (242, 475) |
|  |  | Ips | 98 (74, 109) | 101 (79, 120) | 231 (186, 267) | 199 (147, 257) | 284 (201,334) |
|  |  | P-value | <0.001 | <0.001 | 0.003 | 0.043 | 0.006 |
|  | 250Hz | Con | 45 (29, 61) | 46 (33, 66) | 141.5(107, 187) * | 91 (56, 150) | 144 (104, 216) |
|  |  | Ips | 28 (18, 34) | 25 (22, 37) | 99 (78, 118) | 65 (37, 98) | 111 (76, 143) |
|  |  |  | <0.001 | <0.001 | <0.001 | 0.009 | 0.008 |
|  | 5Hz | Con | 45 (29, 60) | 38 (27, 64) | 96 (49, 118) | 45 (25, 91) | 130 (80,186) |
|  |  | Ips | 20 (16- 28) | 22 (13- 32) | 54 (40- 72) | 33 (16- 71) | 70 (52- 94) |
|  |  | P-value | <0.001 | <0.001 | <0.001 | 0.49 | 0.001 |
| Abbreviations: Con, contralesional side of the stroke lesion; Ips, ipsilesional side of the stroke lesion; Values are in CPT unit (1 unit=0.01mA), and are expressed as median (interquartile range); P- value (contralesional side vs. ipsilesional side) refers to the results of Wilcoxon’s signed-rank test. | | | | | | | |
