# Supplementary material for: Quantitative and Fiber-Selective Evaluation for Central Poststroke Pain
Source: Neural Plast. 2022 Jun 6;2022:1507291. doi: 10.1155/2022/1507291 (PMC9192306; doi:10.1155/2022/1507291)
Supplement: Supplementary Materials — (1) Median CPT parameters of the contralesional side and ipsilesional side in CPSP patients. (2) Pain symptomatology of central poststroke pain. (3) STROBE Checklists. [file 1507291.f1.zip › Supplement 2.docx]

**Table 2.** **Pain symptomatology of central post-stroke pain**

| **Variable** |  | Thalamus  n=13 | Internal capsule  n=44 |
| --- | --- | --- | --- |
| **Pain duration** | ≤1wk | 5(38.46%) | 19(43.18%) |
|  | >1wk, ≤2wk | 3(23.08%) | 12(27.27%) |
|  | >2wk, ≤4wk | 2(15.38%) | 6(13.63%) |
|  | >1 mo, ≤3 mo | 3(23.08%) | 7(15.9%) |
| **Drug management** | Antiplatelet Therapy | 3(23.08%) | 32(72.73%) |
|  | Blood pressure reduction | 10(76.92%) | 30(68.18%) |
|  | Blood-lipoids reduction | 4(30.77%) | 32(72.73%) |
|  | Anticonvulsant Agents | 3(23.08%) | 8(18.18%) |
| **Rehabilitation** | Physical therapy | 10(76.92%) | 35(79.55%) |
|  | Occupational therapy | 11(84.62%) | 37(84.09%) |
| **Type of Pain** | Touch and pressure | 5(38.46%) | 32(72.73%) |
|  | Pin prick | 12(92.31%) | 17(38.64%) |
|  | Temperature | 8(61.54%) | 19(43.18%) |
|  | Vibration | 6(46.15%) | 30(68.18%) |
| **Abnormal sensation**  **localization** | Hemibody | 2(15.38%) | 5(11.36%) |
|  | Upper limb | 8(61.54%) | 14(31.82%) |
|  | Lower limb | 3(23.08%) | 25(56.82%) |
| Abbreviations: n, number; wk, week; mo, month. Data are number of patients (%). | | | |
